# Cellulase activity mapping of *Trichoderma reesei* cultivated in sugar mixtures under fed-batch conditions

**DOI:** 10.1186/1754-6834-6-79

**Published:** 2013-05-17

**Authors:** Etienne Jourdier, Céline Cohen, Laurent Poughon, Christian Larroche, Frédéric Monot, Fadhel Ben Chaabane

**Affiliations:** 1 IFP Energies nouvelles, 1 et 4 avenue de Bois-Préau, 92852 Rueil-Malmaison, France; 2 Clermont Université, Université Blaise Pascal, Labex IMobS3, Institut Pascal, Polytech Clermont-Ferrand, 24 av. des Landais, BP 20206, 63174 Aubière cedex, France

**Keywords:** *Trichoderma reesei*, Sugar mixture, Fed-batch cultivation, Carbon flux limitation, Industrial protocol, Inducer, Cellulase, Xylanase, β-glucosidase, On-site enzyme production, Bioethanol

## Abstract

**Background:**

On-site cellulase production using locally available lignocellulosic biomass (LCB) is essential for cost-effective production of 2^nd^-generation biofuels. Cellulolytic enzymes (cellulases and hemicellulases) must be produced in fed-batch mode in order to obtain high productivity and yield. To date, the impact of the sugar composition of LCB hydrolysates on cellulolytic enzyme secretion has not been thoroughly investigated in industrial conditions.

**Results:**

The effect of sugar mixtures (glucose, xylose, inducer) on the secretion of cellulolytic enzymes by a glucose-derepressed and cellulase-hyperproducing mutant strain of *Trichoderma reesei* (strain CL847) was studied using a small-scale protocol representative of the industrial conditions. Since production of cellulolytic enzymes is inducible by either lactose or cellobiose, two parallel mixture designs were performed separately. No significant difference between inducers was observed on cellulase secretion performance, probably because a common induction mechanism occurred under carbon flux limitation. The characteristics of the enzymatic cocktails did not correlate with productivity, but instead were rather dependent on the substrate composition. Increasing xylose content in the feed had the strongest impact. It decreased by 2-fold cellulase, endoglucanase, and cellobiohydrolase activities and by 4-fold β-glucosidase activity. In contrast, xylanase activity was increased 6-fold. Accordingly, simultaneous high β-glucosidase and xylanase activities in the enzymatic cocktails seemed to be incompatible. The variations in enzymatic activity were modelled and validated with four fed-batch cultures performed in bioreactors. The overall enzyme production was maintained at its highest level when substituting up to 75% of the inducer with non-inducing sugars.

**Conclusions:**

The sugar substrate composition strongly influenced the composition of the cellulolytic cocktail secreted by *T. reesei* in fed-batch mode. Modelling can be used to predict cellulolytic activity based on the sugar composition of the culture-feeding solution, or to fine tune the substrate composition in order to produce a desired enzymatic cocktail.

## Background

The bioconversion of lignocellulosic biomass (LCB) into biofuels or chemicals such as bioethanol requires cellulolytic enzymes, e.g. cellulases and hemicellulases, in order to hydrolyse both cellulose and hemicellulose into their respective monomeric sugars [1]. Cellulases display three main types of enzymatic activities: endo-1,4-β-glucanases cutting cellulose chains internally, exo-1,4-β-glucanases (also called cellobiohydrolases) releasing cellobiose from cellulose chains ends, and β-glucosidases hydrolysing cellobiose to glucose. Hemicellulases or newly discovered oxidative activities may also improve LCB hydrolysis [2]. Owing to its very high secretion capacity, the filamentous fungus *Trichoderma reesei* (teleomorph *Hypocrea jecorina*) has currently been used for the industrial production of cellulolytic enzymes cocktails [3].

### Industrial aspects of cellulolytic enzymes production

Complete cellulose hydrolysis requires substantial cellulase loadings. The supply in enzymes is therefore a key issue for industrial LCB bioconversion. Considering biofuels, on-site cellulase (and hemicellulase) production has often been regarded as an attractive way to limit cellulase production costs, avoiding purification and stabilization of the enzymes produced as well as their transportation. In addition, on-site production allows direct use of the LCB resource available locally. Even in this case, economic studies concluded that much progress was still required to decrease cellulase costs [4-6].

To jointly achieve high productivity and yield at industrial scale, the production of cellulolytic enzymes must be conducted under carbon flux limitation in either fed-batch or continuous mode [7,8]. Using the highly-inducing sugar lactose, the specific production rate of cellulolytic enzymes was around 2.5 fold higher under carbon flux limitation than under carbon excess [9]. Batch cultivation on cellulose could afford a satisfactory carbon flux limitation when cellulose hydrolysis was slower than sugar uptake capacity. However, kinetics of these two unitary reactions are rather complex and difficult to manage [8] as well as stirring and aeration in viscous cellulosic media. In comparison to batch cultivation, fed-batch process with soluble carbon sources has increased the final enzyme concentration by a factor of three and the corresponding productivity by a factor of four [10]. The industrial fermentation process developed by IFPEN consists in a two-phase culture including a first quick cellular growth in excess of substrate and a subsequent cellulase production performed in fed-batch mode under carbon flux limitation [11]. Mixed carbon sources like LCB hydrolysates are potentially usable [12], but their effects on productivity and characteristics of the enzymatic cocktail have never been studied in detail.

### Use of lignocellulosic substrates for enzyme production

Many studies have compared the enzymatic activities produced by *T. reesei* on various substrates including pretreated cellulosic materials ([10,13,14] for reviews on 1980s studies and [15-21] for more recent studies). Steam-pretreated spruce, willow and corn stover were compared to Solka Floc cellulose: differences in specific activities were low for cellulase but significant for β-glucosidase and hemicellulase [16]. Xylanase and mannanase activities were correlated to xylan content in substrate and β-glucosidase activity was found to be 2-fold higher on lactose than on LCB substrates [19]. The induction of enzymatic activities was confirmed by the secretome analysis of cocktails produced on different substrates [17]. However, these studies were performed in batch so that the effects observed on the cocktail characteristics cannot be assuredly extrapolated to industrial fed-batch conditions.

The effect of soluble carbon sources in fed-batch or continuous cultures at the laboratory (2 L) and pilot (3 and 30 m^3^) scales has already been reported [11,12,22,23]. Xylose or glucose culture-feedings led to poor productions of cellulolytic enzymes [12]. The partial addition of lactose to either xylose or hemicellulose hydrolysates feeds led to high enzymes concentrations (around 30 g L^-1^ proteins). Compared to pure lactose feed case, concentrations in β-glucosidase and cellulase were reduced but balanced by a 5 to 10- fold increase in xylanase [12,22]. Addition of xylose in glucose/cellobiose mixture feed improved xylanase activity [23]. The partial substitution of lactose by glucose in the feed led to higher β-glucosidase level. This effect was not confirmed when using glucose-rich hydrolysates [11,12].

In literature, few cultures on three-component sugar mixtures (lactose, xylose and glucose) have been described using purified sugars. It is therefore difficult to deduce the true effect of degradation compounds, like weak organic acids, furan compounds or lignin derivatives that can be found in LCB hydrolysates.

### Aim of the study

The aim of this study was to assess the effect of culture feeding-sugars on the cellulase secretion by *T. reesei*. Cultures were carried out in fed-batch mode with soluble purified carbon sources, using the hyperproducing mutant strain CL847 [24]. To ensure that results could be extrapolated, the study was performed at laboratory scale (for large screening) but under industrial-like conditions (for a valid extrapolation) The effect of sugar mixtures on productivity and cocktail characteristics was modelled. The resulting models were validated with fed-batch cultures performed in bioreactor.

## Results

### Design of the study

**Figure 1:**
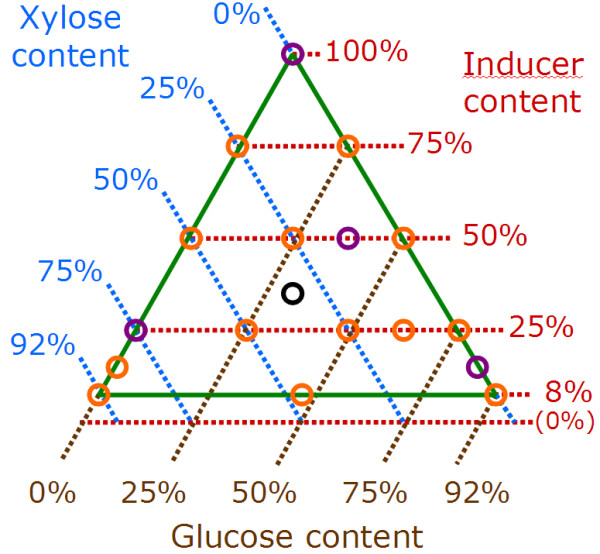
**Schematic view of the mixture design used in the study.** The effect of the feed substrate composition (in fed-batch after cell growth on glucose) was investigated with a 3-factor mixture design which can be represented in a triangle. The studied domain (in green) is defined by the factors ranges: inducer content from 8 to 100% (in red), xylose content from 0 et 92% (in blue), glucose content from 0 to 92% (in brown). For every point in the triangle, the sum of inducer, xylose et glucose contents makes 100%. Circles represent experimental points: mixtures tested in fed-flask protocol to create the models (in orange and purple), mixtures tested in bioreactor to validate the models for lactose inducer (in purple), and central point used to assess protocol reproducibility (in black). This mixture design was repeated twice: once for lactose inducer and once for cellobiose inducer.

Since glucose and xylose are the main constituting monomers of LCB, they were chosen to assess the effect of sugars found in hydrolysates. Since none of them induced cellulase production by *T. reesei*, glucose and xylose mixtures were supplemented with an inducer. Lactose and cellobiose stood as candidates for induction. Lactose was not found in LCB hydrolysates whereas cellobiose might accumulate when LCB hydrolysis was incomplete. A 3-factor mixture (inducer, xylose and glucose) was designed with a minimal inducer content of 8% to allow sufficient induction. To compare the respective inducing effects of lactose and cellobiose, a basal mixture design was applied twice, with lactose and with cellobiose, separately. Figure 1 shows the basal mixture design and the experimental points (fed-batch compositions) tested.

**Figure 2:**
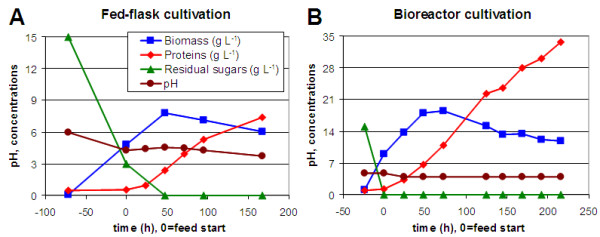
**Monitoring examples for fed-flask and bioreactor cultivations.** pH and concentrations were monitored during fed-flask cultivation (**A**) and bioreactor cultivation (**B**) during growth phase with excess glucose (time before 0 h) then during protein production phase under carbon flux limitation (time after 0 h) by feeding with a sugar mixture solution composed of 50% lactose, 12.5% xylose and 37.5% glucose. In fed-flask protocol pH was stabilized owing to a stoichiometric mix of ammonia in the feed, whereas in bioreactor pH was controlled with ammonia pulses.

To assess the effect of sugar feed composition on both kinetics and characteristics of enzyme secretion, cellulase production was performed using a miniaturized protocol (thereafter called fed-flask protocol) which reproduced in flasks the behaviour of *T. reesei* in bioreactor [9]. This protocol was based on a fed-batch culture in flask with a mixed feed of carbon and nitrogen sources which enabled quasi stable culture pH with minimal equipment. Figure 2 shows a representative example of pH and protein monitoring in a fed-flask culture, compared to the bioreactor culture with identical sugar composition. Kinetics were similar with a stabilization of cell biomass after growth phase then a quasi-linear protein production of cellulolytic enzymes. Bioreactor cultivation yielded higher concentrations because of a higher oxygen transfer but the specific protein production rate was similar in both reactors [9].

### Reproducibility and correlations

The first response used to characterize productivity was the mean value of the specific production rate of protein, calculated over production phase. To characterize the enzymatic cocktails produced, five enzymatic activities were measured in final media. Two of them were global activities of the whole cocktail, i.e. cellulase activity on filter paper [25] and xylanase activity on oat spelts xylan [26]. Three of them were activities specifying the class of cellulolytic enzymes i.e. endoglucanase activity on Carboxymethyl cellulose [25], cellobiohydrolase activity due to Cel7A/CBH I on *p*NPL and β-glucosidase activity on *p*NPG.

With 8 runs (fed-batch mixture compositions) by series, 6 series were necessary to perform all cultivations. The reproducibility of the whole experimental design (cell growth, cellulase production in fed-flask protocol, analyses) was verified on the central point of the mixture design with lactose inducer (fed-batch containing 1/3 lactose + 1/3 xylose + 1/3 glucose, black circle Figure 1), by repeating this condition 10 times (3 or 4 times in 3 different series). Reproducibility was satisfactory, with residual standard deviations lower than 10% for 5 of 6 enzyme activities, and 16% for xylanase activity (Table 1). The standard deviation of each measurement was lower than the variations observed thereafter, which validated the experimental design.

**Table 1:** Reproducibility of the experimental design

**Measurement**	**Unit**	**Mean value**	**Std. dev.**	**(RSD)**
Specific protein production rate	mg_P_ g_X_^-1^ h^-1^	11.9	± 1.0	(± 9%)
Specific cellulase activity	IU mg_P_^-1^	0.47	± 0.06	(± 10%)
Specific xylanase activity	IU mg_P_^-1^	130	± 20	(± 16%)
Specific endoglucanase activity	IU mg_P_^-1^	11.2	± 0.5	(± 4%)
Specific Cel7A (CBH I) activity	IU mg_P_^-1^	0.23	± 0.02	(± 8%)
Specific β-glucosidase activity	IU mg_P_^-1^	0.52	± 0.06	(± 10%)

The reproducibility of the whole experimental protocol (cell growth + protein production in fed-flask protocol + analyses) was assessed on the central point of the mixture design with lactose inducer (black circle Figure 1), by 10 repeats in 3 different series.

**Figure 3:**
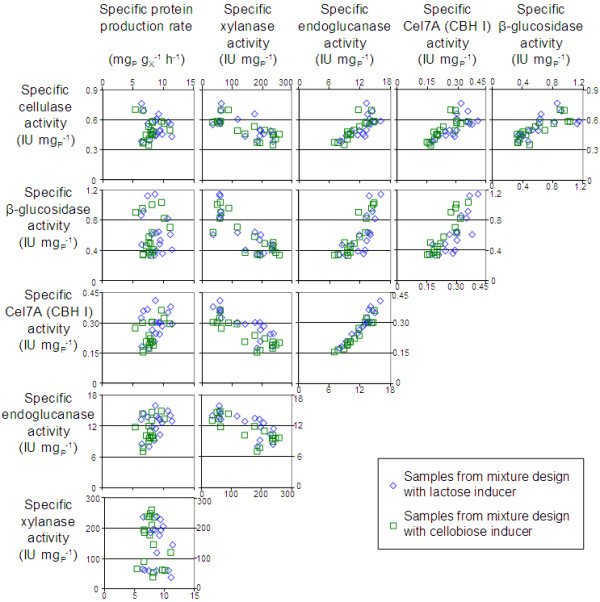
**Correlation diagram for the 6 measured responses.** Raw values of the 6 responses (specific protein production rate and 5 specific enzymatic activities) measured in samples from mixture designs with lactose inducer (blue diamonds) or with cellobiose inducer (green squares) are plotted one against each other.

The correlations between the six responses using raw values measured in both mixture designs are shown in Figure 3. No direct correlation was found between the specific protein production rate and each specific enzymatic activity (left column Figure 3). Therefore, productivity and cocktail characteristics were two independent results in fed-batch cultures. The four individual cellulolytic activities exhibited positive correlations between each other. As already shown [25], filter paper activity resulted from the combined activities of endoglucanases, cellobiohydrolases and β-glucosidase, The highest correlation was observed between endoglucanase activity and Cel7A(CBH I) activity. In contrast, xylanase activity showed a negative correlation with the 4 other cellulolytic activities.

### Resulting models

Raw values for the 6 studied responses and the 2 mixture designs were modelled as a function of the 3 factors (inducer, xylose and glucose contents) using a quadratic model:

$$\begin{array}{l} Y=a\left[\mathrm{Ind}\right]+b\left[\mathrm{Xyl}\right]+c\left[\mathrm{Glu}\right]+d\left[\mathrm{Ind}\right]\left[\mathrm{Xyl}\right]\\ \;+e\left[\mathrm{Ind}\right]\left[\mathrm{Glu}\right]+f\left[\mathrm{Xyl}\right]\left[\mathrm{Glu}\right] \end{array}$$

where [Ind], [Xyl] and [Glu] are the sugar contents (mass fraction of total sugars) for inducer, xylose and glucose respectively. When the model terms d, e or f were not significant, they were removed from the model only when it increased the predicted-R^2^ (In Design Expert, predicted-R^2^ assesses the predictive capacity of a model, independently of its number of terms). The terms values and statistical analyses for the 12 models are available in Additional file 1.

All models were significant (p-value < 0.05), with predicted-R^2^ between 0.45 and 0.92 (a negative predicted-R^2^ would imply that the overall mean value is a better predictor than the model, which was not the case here), and with standard deviations in the same range than the experimental ones observed during reproducibility assessment. In a mixture design, model terms values are hardly analysable directly since factors are not independent (their sum is constant). The effect of each sugar was better explained in contour plots representing the significant models.

### Effect of sugar mixture on production kinetics

**Figure 4:**
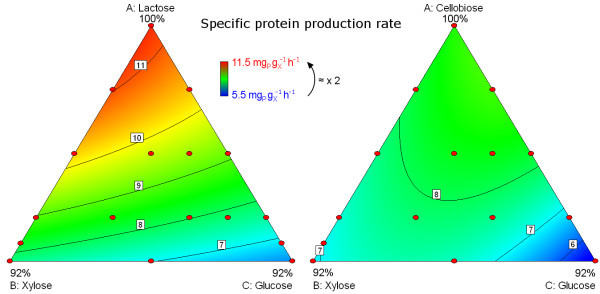
**Effect of sugar mixtures on specific protein production rate.** Specific protein production rate was measured during cellulase production with fed-batch process in fed-flask protocol for both mixture designs with lactose inducer (left) and cellobiose inducer (right) then modelled using a quadratic model (Additional file 1). Mixture compositions are shown in triangles according to Figure 1. Model values are in colour scale from blue (low values) to red (high values) according to the legend. Intermediate values are shown by contour lines (black lines). Red dots are experimental points used for the models.

Figure 4 shows the effect of the fed sugar mixtures on the specific protein production rate measured in fed-flask protocol for both mixture designs with either lactose inducer (left) or cellobiose inducer (right).

The main effect was due to inducer content which increased the specific protein production rate. Lactose had a stronger effect than cellobiose (higher specific rate at high content in feed). However at an identical inducer content (horizontal line in Figure 1), specific protein production rate was higher when glucose was replaced by xylose (moving from right to left in each triangle). Therefore, xylose had a lower repressing effect on enzyme production than glucose.

Specific protein production rate was 5.5 to 11.5 mg_P_ g_X_^-1^ h^-1^ with lactose as an inducer versus 5.5 to 8.9 mg_P_ g_X_^-1^ h^-1^ for cellobiose. Thus, the 92% replacement of the inducer by a non-inducing sugar preserved at least 50% of the specific productivity.

### Effect of sugar mixture on cocktail characteristics

**Figure 5:**
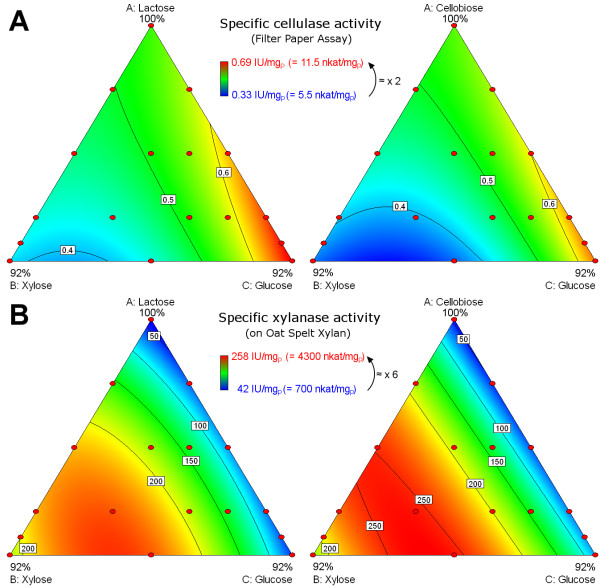
**Effect of sugar mixtures on global enzymatic activities.** Cellulase (**A**) and xylanase (**B**) activities were measured in final enzymatic cocktails produced in fed-flask protocol for mixture designs with either lactose inducer (left) or cellobiose inducer (right). They were modelled using a quadratic model (Additional file 1). Representation is identical to Figure 4.

The effects of the fed sugar mixtures on global enzymatic activities (cellulase and xylanase activities) are shown in Figure 5. For both activities, the inducer choice had little influence and the effects of sugar mixtures were similar for lactose and cellobiose inducers.

Low variations were observed for specific cellulase activity in the overall mixture design, with a 2-fold variation (0.33 IU mg_P_^-1^ to 0.69 IU mg_P_^-1^). The main effect was due to xylose content in the feed which lowered cellulase activity. Surprisingly, glucose had beneficial effect and maximal cellulase activities were found when feedings were mostly-containing glucose (bottom right apex). Specific cellulase activity was slightly higher with lactose than with cellobiose as an inducer.

High variations were observed for specific xylanase activity, with a 6-fold variation (40 IU mg_P_^-1^ to 260 IU mg_P_^-1^). Variations were only linked to xylose content. Specific xylanase activity increased linearly when xylose content varied from 0 to 30%, then was almost constant up to 92% xylose. Specific xylanase activity was slightly higher when using cellobiose rather than lactose as an inducer.

**Figure 6:**
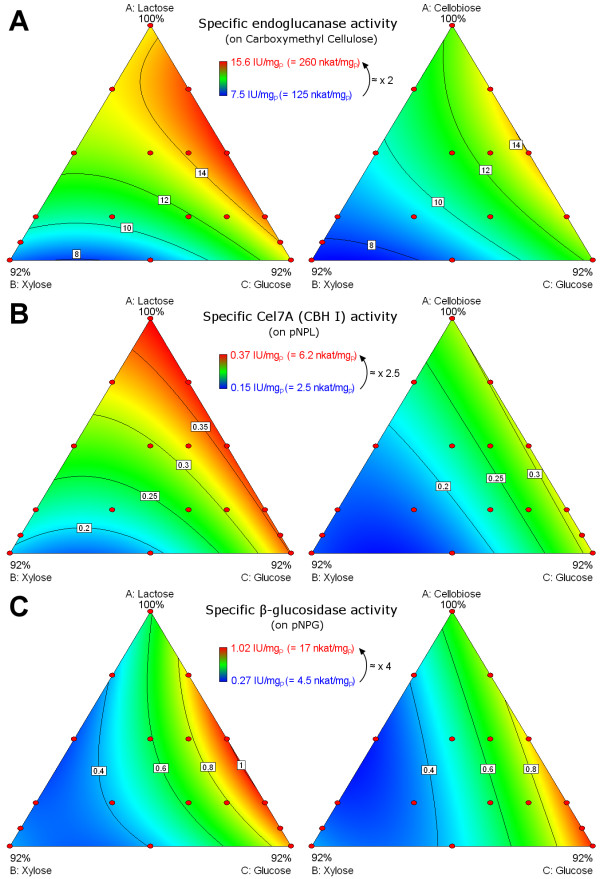
**Effect of sugar mixtures on particular cellulolytic activities.** Endoglucanase (**A**), cellobiohydrolase activity due to Cel7A(CBH I) (**B**), and β-glucosidase (**C**) were measured in final enzymatic cocktails produced in fed-flask protocol for both mixture designs with either lactose inducer (left) or cellobiose inducer (right) then modelled using a quadratic model (Additional file 1). Representation is identical to Figure 4.

Figure 6 shows the effect of fed sugar mixtures on the three enzymatic activities needed to hydrolyse cellulose i.e. endoglucanase, cellobiohydrolase, and β-glucosidase. As observed for global cellulase activity, the effects of sugars were similar with each inducer. Enzymatic activities were however slightly higher with lactose than with cellobiose. Maximal variations were 2-fold for endoglucanase, 2.5-fold for cellobiohydrolase Cel7A (CBH I), and 4-fold for β-glucosidase. As for global cellulase activity, the main effect was due to xylose that lowered the three activities. In particular the specific activity of β-glucosidase was divided by two when xylose in the feed exceeded 30%.

The maximum activity was reached for different substrate compositions: at high xylose content for xylanase, and at low xylose content for the four cellulolytic activities for which the maximum was reached at different glucose/inducer ratio, depending also on the inducer choice.

### Validation of the model by cultures in bioreactor

**Figure 7:**
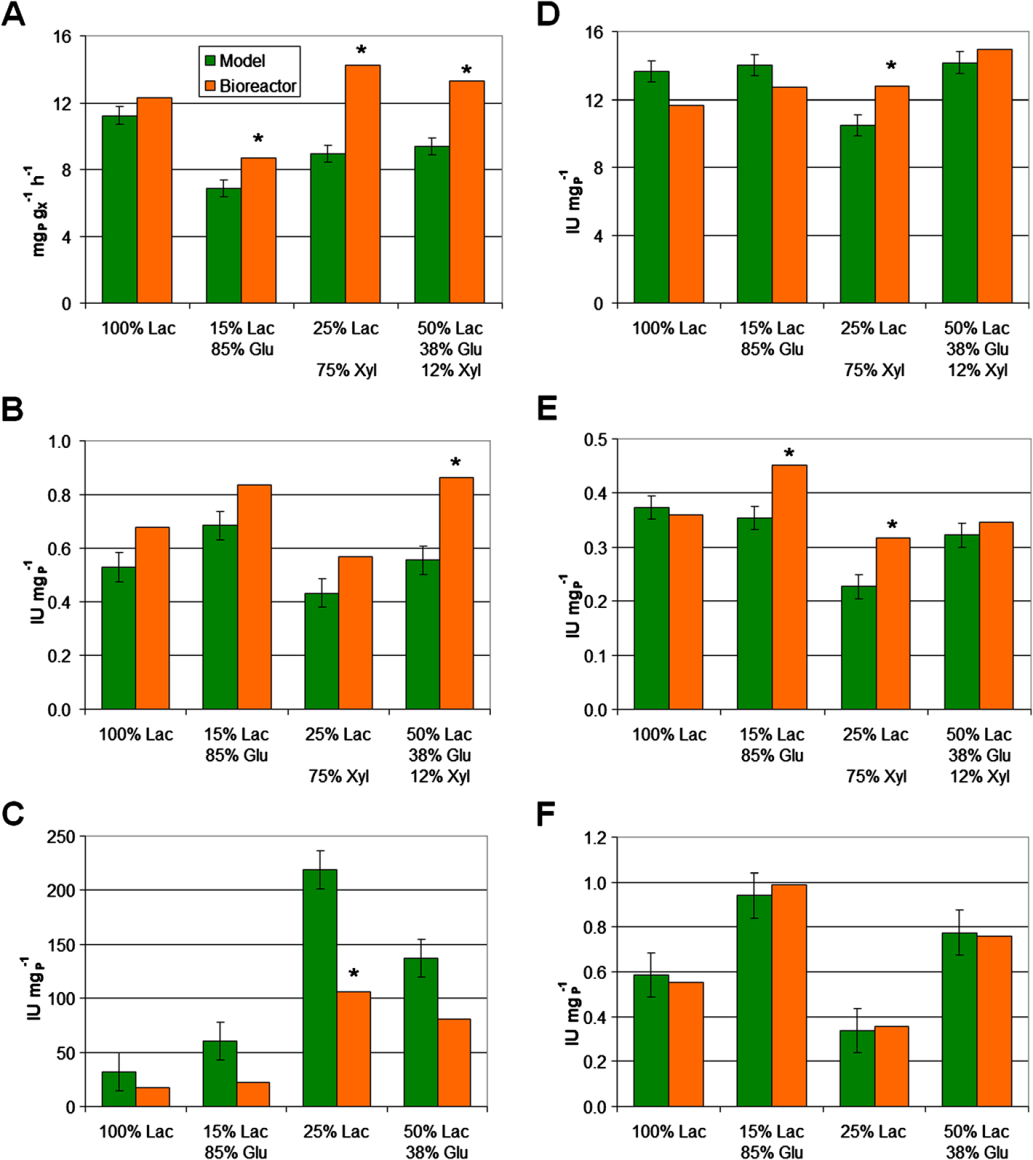
**Models validation with bioreactor cultivations.** Four fed-batch bioreactor cultivations, with 4 different feed compositions (on the abscissa), were performed to validate the models for (**A**) specific protein production rate, (**B**) specific cellulase activity, (**C**) specific xylanase activity, (**D**) specific endoglucanase activity, (**E**) specific Cel7A(CBH I) activity and (**F**) specific β-glucosidase activity. Green bars represent predicted values using models, and orange bars represent values from bioreactor cultivations. Error bars are models standard deviations. Stars indicate bioreactor values significantly different from the model (outside the prediction interval with 99% confidence).

In order to validate the models obtained in fed-flask protocol, four cultivations were performed in bioreactors fed with distinct sugar mixtures. Since lactose had a higher inducing effect than cellobiose, the 4 feeding mixtures were supplemented with lactose (Figure 1). An example of pH and concentrations monitoring is shown in Figure 2 for a feed composed of 50% lactose, 12.5% xylose, 37.5% glucose. The six responses corresponding to the four bioreactor cultivations were compared to the model predictions (Figure 7).

Regarding the specific protein production rate (Figure 7A), the model accurately predicted the value for 100% lactose feeding, as previously observed [9]. Productivity for xylose-containing feeds was higher in bioreactor than the one predicted by the model, which is positive for scaling-up. Regarding global enzymatic activities (cellulase and xylanase, Figure 7B and C), the trends observed in bioreactor were consistent with those predicted by the models. The enzymatic activities were accurately predicted by the model for three conditions out of four. Regarding the three particular cellulase activities (Figure 7D to F), the models were appropriate to predict the enzymatic activities in bioreactor cultivation, especially for β-glucosidase activity (Figure 7F).

## Discussion

### Efficiency of the design

The fed-flask protocol used in this study allowed 34 fed-batch cultivations (plus 10 cultivations for reproducibility assessment), which would have been tedious in bioreactors, because cultivation is time-consuming (10 days). Since simple flasks were used, growth phases of a series could be performed in parallel with feeding phases of another series. Suitable reproducibility was obtained and cultivations in bioreactors validated the specific activities expected.

The lower specific protein production rate observed in fed-flask protocol may be due to diffusion or transfer issues which modified metabolism. Feeding rate was very low in fed-flask protocol so that feeding was not really continuous but rather intermittent, and mixing was less efficient. These combined effects may create higher local sugar concentrations which may result in lower protein induction.

The ranges of enzymatic activities obtained were higher in this study than previously reported, with 2 to 4- fold variations for cellulase activities versus 50% when comparing different lignocellulosic materials [16,19]. This may be due to the mixture design chosen, which allowed the comparison of much more different substrate compositions.

### Effect of inducer nature on enzyme secretion

Surprisingly, similar patterns were observed for lactose and cellobiose inducers, although i) cellobiose was generally considered as a weaker inducer than lactose, and ii) lactose-induced cellulase involved lactose assimilation and metabolism of the resulting galactose moiety [27]. The lower cellulase production on cellobiose has generally been attributed to inhibitory actions such as catabolite repression by the glucose released from extracellular cellobiose hydrolysis [28], but this effect is negligible in glucose-derepressed strains like the one used in this study. Cellulase induction by cellobiose only occurred when its uptake was more favourable than extracellular hydrolysis [29], or when a di-glucoside permease with higher affinity than that of β-glucosidase was active [30]. Recently, intracellular β-glucosidases were shown to be involved in cellulase induction by cellobiose [31]. A similar mechanism was observed for cellulase induction by lactose, with probable involvement of a lactose permease [32]. In both cases, inducer uptake was required for induction, which occurred preferentially under carbon flux limitation, as it was the case in this study. The differences in specific protein production rate between lactose and cellobiose may be due to differences in extracellular hydrolysis or uptake rates, which would modify the induction signal. In any case, the fact that similar enzymatic patterns were observed for cellobiose and lactose may indicate that, in a glucose de-repressed background, both compounds trigger a common induction mechanism.

### Correlations and incompatibilities between enzymatic activities

High correlations were observed between the three particular cellulase activities, especially between endoglucanase and cellobiohydrolase activities (Figure 3). Since these activities are required for the complete cellulose hydrolysis, the coordinate regulation of these enzymes at transcriptional level is very likely [33]. In contrast, xylanase activity was negatively correlated with the four cellulolytic activities. At low specific growth rate, protein production was shown to be limited by secretion [34]. Therefore competition for secretion may occur between cellulase and xylanase enzymes.

Among the factors affecting the five studied activities, the xylose content was the main one. Xylose had a positive impact on xylanase activity but a negative impact on all the other cellulase activities. This result had previously been observed with lignocellulosic materials (pretreated or hydrolysed) [12,16,19,22], but it was not clear whether sugar composition or occurrence of LCB degradation compounds prevailed. Our study showed that sugar composition accounted for the main effects. Therefore, the enzymatic composition of a cocktail can be predicted from any substrate using the models.

### Relevance of xylanase and β-glucosidase activities for hydrolysis

Xylanase and β-glucosidase activities exhibited the highest variations, with a 6-fold increase in xylanase activity correlated with a 4-fold decrease of β-glucosidase activity. Accordingly, high levels of these two activities seemed rather incompatible in the enzymatic cocktails produced by *T. reesei*.

The importance of hemicellulase activities to improve LCB hydrolysis has been extensively studied in the last 5 years, either to understand biochemical mechanisms [35-44] or to design optimal enzyme mixtures [45-48]. All these studies concluded that hemicellulase activitiy had a beneficial effect on xylose and glucose release, the extent of the gains being highly dependent on the raw materials, the pretreatment and the hemicellulase activity.

Besides, the beneficial effect of β-glucosidase activity on cellulose hydrolysis has been shown for years. β-glucosidases are inhibited by the end-product glucose [49], and the β-glucosidase content is *T. reesei* enzymatic cocktails is often low [50], limiting the cellulose hydrolysis rate because of accumulation of cellobiose, a potent inhibitor of cellobiohydrolases.

If both activities are actually required for efficient LCB hydrolysis, a compromise will have to be reached in the choice of the substrate in order to get an optimal enzymatic cocktail. Since β-glucosidase content is very low in *T. reesei* cocktail, increasing β-glucosidase at the expense of xylanase activity will probably be the favourite choice for cellulase production, but it will limit the choice of the substrate feed composition.

With genetic and enzymatic engineering approaches, many work has been done to increase the β-glucosidase activity in *T. reesei* cocktail, by over-expressing the native β-glucosidase [51,52], heterologous β-glucosidases [53-56] including a highly improved β-glucosidase engineered by L-shuffling [57]. The resulting strains will probably have different enzymatic patterns, especially for β-glucosidase activity, which will modify the optimized substrate composition. Testing genetic engineered strains with similar experimental protocol may be very interesting to understand regulation effects in industrial-like fed-batch conditions.

## Conclusion

The effect of sugar mixtures as a substrate for celluase production by a glucose-derepressed and cellulase-hyperproducing mutant strain of *T. reesei* was assessed in simulated industrial fed-batch conditions. Owing to the miniaturized "fed-flask" protocol, 34 different mixtures were tested, for both inducers lactose and cellobiose. Enzymatic activity mappings were modelled and validated by bioreactor cultivations.

Using these models, the enzymatic composition of a cocktail can be predicted depending on the substrate composition, for example for xylose-rich hemicellulosic hydrolysates or glucose-rich cellulosic hydrolysates, and depending on inducer content. Conversely, these models can be used to choose the correct substrate composition for a desired enzymatic cocktail, to favour an enzymatic activity at the expense of an other, as it will be necessary for the incompatible xylanase and β-glucosidase activities.

## Methods

### Strain and culture media

*T. reesei* CL847 is a glucose-derepressed and cellulase-hyperproducing strain obtained from QM 9414 strain by several steps of mutagenesis and selection, from Cayla Company, Toulouse, France [24]. Its behaviour and performances were comparable to other hyperproducer strains like e.g. Rut-C30 [14]. Spores were conserved in cryotubes at −80°C with 50% glycerol.

Culture media for fed-flask protocol, preculture, and bioreactor cultivations were prepared according to [9].

### Fed-Flask cultivations

Fed-flask cultivations were performed according to [9] with few modifications. A shared growth phase was performed each week for 8 fed-flasks. For each, 2 Fernbach flasks were prepared with 250 mL medium culture supplemented with 15 g L^-1^ glucose. Both flasks were inoculated with around 10^6^ spores then incubated at 30°C and 150 rpm in an Infors rotary shaker. After 72 h, the two flasks were mixed then split in 8 wide neck 250 mL Erlenmeyer flasks with 50 mL broth per flask. These flasks were incubated in the same conditions and fed at 0.3 mL h^-1^ average rate, using calibrated peristaltic pumps (Dasgip MP8) performing periodic additions at a rate close to 1 mL h^-1^. The feed solution composition was: total sugars 50 g L^-1^; NH_3_ 20% (11 N) 15 mL L^-1^; (NH_4_)_2_SO_4_ 0.8 g L^-1^. This composition was calculated from the stoichiometry of protein production to meet carbon, nitrogen and sulphur requirements, and stabilize pH [9].

### Bioreactor cultivations

Bioreactor cultivations were carried out in Dasgip fedbatch-pro bioreactors with an initial working volume of 750 mL. A shared preculture was performed in a Fernbach flask with 250 mL flask medium culture, inoculated with around 10^6^ spores, incubated 72 h at 150 rpm and 30°C in an Infors rotary shaker, then split in four for the four bioreactors. Growth phase in batch was performed on 15 g L^-1^ glucose at pH 4.8 and 27°C for 24 h. Then fed-batch was performed at pH 4.0 and 25°C with feeding at 2 mL h^-1^ by a 250 g L^-1^ mixed sugars solution. pH was automatically adjusted with 5.5 N NH_4_OH solution. Aeration rate was fixed at 30 sL h^-1^ and agitation was regulated to maintain dissolved oxygen at minimum 40% of its concentration at saturation.

### Analytical measurements

Culture medium was filtrated using Whatman GF/C filters. For biomass concentration determination, biomass cake was washed with distilled water then dried at 105°C until constant weight.

Protein concentration was measured in supernatants against BSA standards (0–1.5 g L^-1^ range with second-order regression) by Lowry method [58] using *DC*™ Protein Assay (Biorad). Lowry method has been recommended by IUPAC [25] and was shown consistent with carbon balance [9] for *T. reesei* cellulases.

Sugars concentration was measured by HPLC. Separation was carried out using Varian Metacarb 87P column with mobile phase milliQ water at 0.4 mL min^-1^, 80°C and pressure around 32 bar; detection was carried out with Waters 2414 refractive index detector.

### Enzyme activity assays on complex polymeric substrates

Reducing sugars released in enzymatic assays were measured with DNS reagent [59], by adding 1.5 or 2 volumes of DNS reagent in samples containing around 1 g/L reducing sugars. The mix was boiled for 5 minutes then diluted with water to reach spectrophotometer linear range. Concentrations equivalents were calculated with a glucose scale by absorbance at 550 nm (global cellulase activity, endoglucanase activity) or with a xylose scale by absorbance at 540 nm (xylanase activity).

Global cellulase activity was measured using the IUPAC standard Filter Paper Assay [25], after a 15-fold miniaturization similar to [60] which allowed working in 2 mL Eppendorf tubes. In order to surround the desired 4% conversion yield, four enzyme dilutions were tested for each sample. This miniaturized protocol was validated by comparison with the standard IUPAC protocol.

Xylanase activity was measured according to IUPAC recommendations [26] on oat spelts xylan (Sigma) in 2 mL Eppendorf tubes. Hundred μL of 2% oat spelts xylan solution was mixed with 100 μL sample dilution (both in 50 mM pH 4.8 citrate buffer), and incubated 10 minutes at 50°C and 600 rpm in Thermomixer (Eppendorf). Reducing sugars were revealed with 300 μL DNS and 5 minutes boiling. Absorbance was measured at 540 nm after dilution with 1.5 mL water. Xylose equivalent concentration was calculated by comparison with a xylose scale, with subtraction of the xylose released in a substrate blank without enzyme. The measure was validated if enzymatic xylose release was in the linear range of the hydrolysis determined at first: below 6% conversion yield (0.7 g/L xylose concentration) in our experimental conditions.

Endoglucanase activity was measured according to IUPAC standard protocol on Carboxymethyl cellulose [25] after a 10-fold miniaturization which allowed working in 2 mL Eppendorf tubes. Incubation was performed in Thermomixer (Eppendorf) at 900 rpm. In order to surround the desired 0.5 g/L glucose equivalent release, four enzyme dilutions were tested for each sample.

### Enzyme activity assays on chromogenic substrates

Both cellobiohydrolase and β-glucosidase activities were measured on chromogenic substrates, *p*NPL and *p*NPG respectively, which release 4-nitrophenol (*p*NP). Assays were performed in 1.5 mL Eppendorf tubes, with 30 minutes incubation at 50°C.

Cellobiohydrolase activity due to Cel7A(CBH I) was measured on *p*NPL. Purified Cel6A (CBH II) has no activity on *p*NPL (Y. Benoit and A. Margeot, personal communication). In our cocktails, we verified that around 90% of the activity on *p*NPL was due to Cel7A by performing cellobiohydrolases inhibition with cellobiose. 50 μL *p*NPL solution at 3 mg/mL (6.5 mM) was mixed with 50 μL sample dilution (both in citrate buffer 50 mM pH 4.8).

β-glucosidase activity was measured on *p*NPG, by mixing 90 μL *p*NPG solution at 1.5 mg/mL (4.8 mM) and 10 μL sample dilution (both in citrate buffer 50 mM pH 4.8).

In both cases, released *p*NP was revealed by adding 100 μL of 2% Na_2_CO_3_ solution. Absorbance was measured at 410 nm and released *p*NP concentration was calculated by comparison with a *p*NP scale from 25 to 200 μM. The measure was validated if released *p*NP was in the scale range.

All enzymatic activities were expressed as specific activities in IU mg_P_^-1^ ( IU meaning μmol min^-1^) and converted in nkat mg_P_^-1^ in the figures legends (1 nkat = 0.06 IU).

### Statistical analyses

Mixture designs analyses were performed using Design Expert v8.0 (Stat-Ease, Inc.).

## Abbreviations

LCB: Lignocellulosic biomass; pNP: 4-nitrophenol; pNPG: 4-nitrophenyl-β-D-glucopyranoside; pNPL: 4-nitrophenyl-β-D-lactopyranoside; CMC: Carboxymethyl cellulose; RSD: Residual standard deviation; SPPR: Specific protein production rate.

## Competing interests

The authors declare that they have no competing interests.

## Authors’ contributions

EJ and FBC designed the study. EJ carried out the experiments and drafted the manuscript. CC performed the bioreactor cultivations. FM supervised the study. CL and LP are academic supervisors of EJ. All authors revised, read and approved the final manuscript.

## Supplementary Material

Additional file 1 **Terms values and statistical analyses for the 12 models.** For each inducer and each response, the values for the 6 terms of the quadratic model are shown next to the statistical analyses of the model (predicted R^2^, standard variation and p-value). Click here for file
