# Supplementary material for: Cellulase activity mapping of Trichoderma reesei cultivated in sugar mixtures under fed-batch conditions
Source: Biotechnol Biofuels. 2013 May 17;6:79. doi: 10.1186/1754-6834-6-79 (PMC3700819; doi:10.1186/1754-6834-6-79)
Supplement: Additional file 1 — Terms values and statistical analyses for the 12 models. For each inducer and each response, the values for the 6 terms of the quadratic model are shown next to the statistical analyses of the model (predicted R2, standard variation and p-value). [file 1754-6834-6-79-S1.pdf]

Table S1 – Terms values and statistic analyses of the models

Terms values for the quadratic models Y = a [Ind] + b [Xyl] + c [Glu] + d [Ind] [Xyl] + e [Ind] [Glu] + f [Xyl] [Glu] and statistic results (predicted R², standard variation and p-value) are presented for the 2 inducers and the 6 studied responses. Empty cells are insignificant terms removed from the models. Terms units are $mg_P\ g_X^{-1}\ h^{-1}$ for specific protein production rate (SPPR) and IU mg_P^{-1} for specific activities.

Inducer	Response	Terms values						Statistic analysis		
		a	b	c	d	e	f	pred. R²	std. dev.	p-value
Lactose	SPPR	11.2	7.08	5.69	4.39	2.63		0.86	0.51	<0.0001
	cellulase	0.53	0.40	0.71			-0.57	0.63	0.054	0.0001
	xylanase	32.5	159	42.6	486	151	568	0.92	17.6	<0.0001
	endoglucanase	13.6	7.4	12.8	8.3	8.8	-11.3	0.85	0.64	<0.0001
	Cel7A(CBH I)	0.37	0.18	0.35			-0.38	0.86	0.022	<0.0001
	β-glucosidase	0.59	0.44	0.83	-0.73	1.15	-1.14	0.78	0.10	<0.0001
Cellobiose	SPPR	8.69	6.76	4.38		6.77	6.06	0.45	0.61	0.0036
	cellulase	0.54	0.36	0.66			-0.69	0.76	0.042	<0.0001
	xylanase	42.6	140	79.6	628		597	0.86	25.2	<0.0001
	endoglucanase	12.3	7.2	11.7		8.07	-6.94	0.71	0.79	<0.0001
	Cel7A(CBH I)	0.30	0.17	0.32	-0.19		-0.33	0.74	0.026	<0.0001
	β-glucosidase	0.68	0.46	1.02	-1.11		-1.28	0.83	0.082	<0.0001
